# Supplementary material for: Excessive abundance of common resources deters social responsibility
Source: Sci Rep. 2014 Feb 24;4:4161. doi: 10.1038/srep04161 (PMC3932447; doi:10.1038/srep04161)
Supplement: Supplementary Information [file srep04161-s1.pdf]

Excessive abundance of common resources deters social responsibility

Supplementary Information

Xiaojie Chen[1,*] and Matjaž Perc[2,†]

[1]*Evolution and Ecology Program, International Institute for Applied Systems Analysis (IIASA), Schlossplatz 1, A-2361 Laxenburg, Austria*

[2]*Faculty of Natural Sciences and Mathematics, University of Maribor, Koroška cesta 160, SI-2000 Maribor, Slovenia*

To verify the robustness of the main results presented in the paper, this supplementary information is devoted to the study of several variations of the proposed collective-risk social dilemma. In particular, we study the effects of the population size (§I), the topology of the population structure (§II), different uncertainties by strategy adoptions (§III), the delay in individual strategy updating (§IV), the birth-death update rule (§V), as well as the effects of cooperator's priority towards limited endowments (§VI). In general, our results remain valid under all considered circumstances.

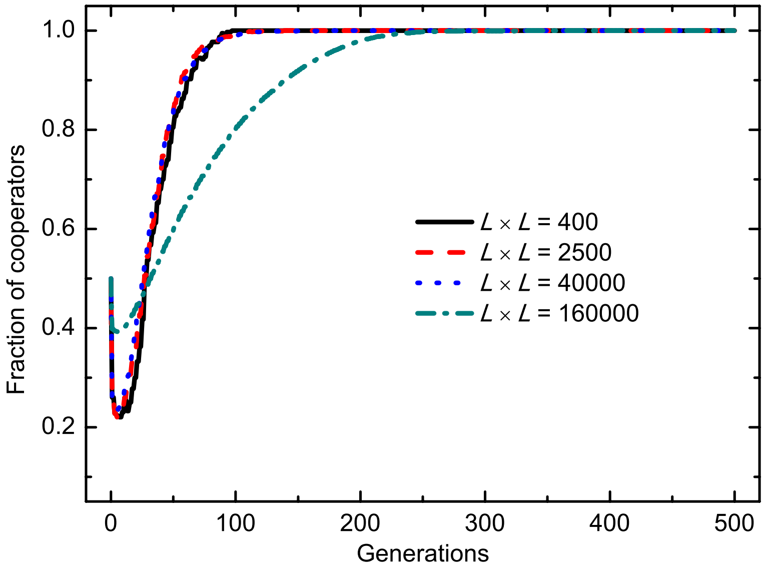

FIG. 1: Time evolution of the fraction of cooperators for different population sizes (see legend). Parameter values are: $b = 10$, $R = 10$ and $\alpha = 10$.

*Electronic address: chenx@iiasa.ac.at

†Electronic address: matjaz.perc@uni-mb.si

Population size

In this section, we present the time evolution of the fraction of cooperators for different population sizes, to see how the outcome of the evolutionary process depends on this quantity. Figure 1 shows that increasing the population size does increase the time for the system to reach the stationary state, but it does not affect the composition of the strategies. For larger population sizes, the system simply needs longer to reach the stationary state.

Population structure

In this section, we consider the studied collective-risk social dilemma on a random network, on the regular ring, and on the square lattice with Moore neighborhood. On a random network, each individual forms a group with other $G-1$ individuals randomly chosen from the whole population, and gets its payoff only from the interactions within the group. While in other interaction networks, each individual participates in all the G groups that are centered not only on itself, but also on its nearest neighbors. Figure 2 shows the stationary fraction of cooperators in dependence on α and R. It can be observed that there exist intermediate α values maximizing the fraction of cooperators, and there exists an upper bound value of R beyond which cooperators die out. This indicates that our findings are robust against the changes in the structure of the interaction networks.

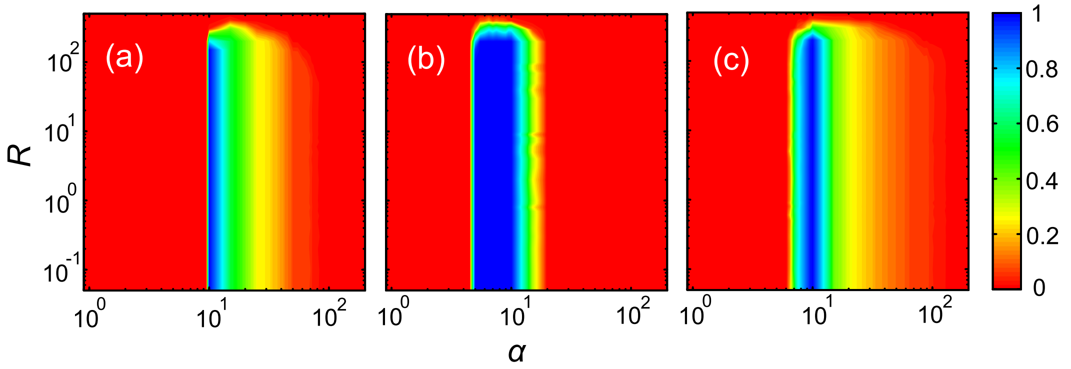

FIG. 2: The stationary fraction of cooperators in dependence on the multiplication factor and the initial amount of common resources, as obtained for different population structures: (a) random network with group size $G = 5$, (b) regular ring with group size $G = 5$, and (c) square lattice with Moore neighborhood ($G = 9$). Parameter values are: $b = 10$, $K = 0.5$, and the population size is 10^4 in all three cases.

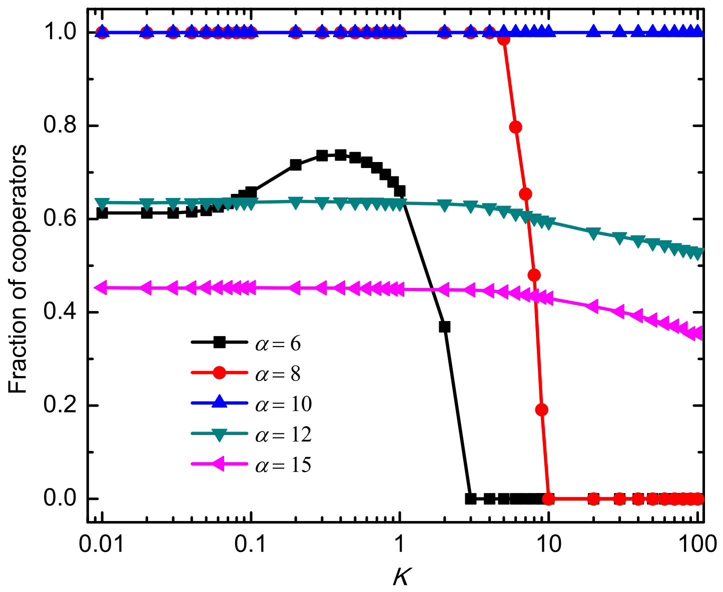

FIG. 3: The stationary fraction of cooperators in dependence on the uncertainty parameter K for different values of the multiplication factor α, as obtained on a square lattice with von Neumann neighborhood. Parameter values are: $b = 10$, $K = 0.5$, and the size of the square lattice is $L^2 = 10^4$.

Uncertainty by strategy adoptions

In this section, we demonstrate the effects of different uncertainty by strategy adoptions on the evolution of cooperation in the studied collective-risk social dilemma. From results presented in Fig. 3 it follows that the stationary fraction of cooperators varying with K displays four different types of behavior, depending on the value of the multiplication factor α. First, for a relatively small value of α, the fraction of cooperators first increases slowly until reaching the maximum value, and then decreases dramatically to zero with increasing K. Second, for a slightly larger α value, full cooperation can be achieved when K varies from 0.01 to 5. But the fraction of cooperators dramatically decreases to zero with further increasing K from 5. Third, for an appropriately intermediate α value, full cooperation can always be achieved when K varies in a large range $[0.01, 100]$. Fourth, for a much larger α value, the fraction of cooperators declines slightly with increasing K. This qualitatively different behavior has been revealed in previous works, and here we demonstrate again that the uncertainly by strategy adoptions plays an important role by the evolution of cooperation [1, 2].

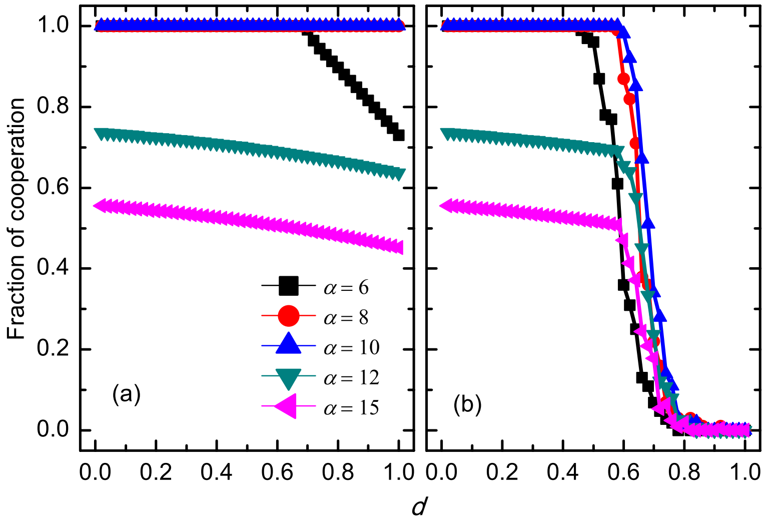

FIG. 4: The stationary fraction of cooperators in dependence on the delay parameter for individual strategy update for different values of the multiplication factor α on a square lattice with von Neumann neighborhood. Here, $b = 10$ and the size of the square lattice is $L^2 = 10^4$. The initial amount of common resources R is 10 in (a) and 500 in (b).

Delay in individual strategy updating

In this section, we consider that each individual is subject to delay during strategy updating. While as before, an individual has the opportunity to imitate the strategy of one randomly chosen neighbor y, the probability for this step to be attempted is $d < 1$ (rather than $d = 1$, as in the main paper). On the other hand, the amount of common resource in each group is still updated at each time step. Figure 4 shows the stationary fraction of cooperators as a function of d for different intermediate values of α. For small initial $R = 10$ in panel (a), the fraction of cooperators varying with d displays three different types of behavior, depending on the value of the multiplication factor α. First, for a relatively small α value, full cooperation is achieved for the delay factor $d < 0.70$. But with increasing d from 0.70, the fraction of cooperators decreases quickly. Second, for slightly larger α values, full cooperation can always be achieved, irrespective of the value of d. Third, for much larger α values, the fraction of cooperators gradually decreases with increasing d. In addition, for much small or much large values of α, full defection is always achieved, irrespective of the value of d (not shown here). For large initial $R = 500$ in panel (b), we see that the fraction of cooperators first decreases slowly with increasing d, and then dramatically decreases to zero after d reaches a critical value, and that this is the case for several different

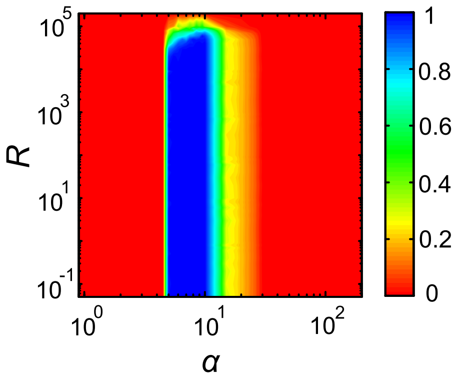

FIG. 5: The stationary fraction of cooperators in dependence on the multiplication factor α and the initial amount of common resources R, as obtained with the birth-death update rule on a square lattice. Here, $b = 10$, $w = 0.5$, and the linear population size is $L = 20$.

intermediate values of α.

Birth-death update rule

With the motivation to consider the studied collective-risk social dilemma in a perhaps biologically more relevant context, we consider the birth-death rule instead of the imitation rule used in the main text. Under the birth-death rule, an individual is chosen for reproduction proportional to fitness at each time step, and then the offspring replaces a random neighbour. Because the fitness has to be positive, following previous works [3, 4], we define an individual x's fitness as $f_x = exp[wP_x]$, where w $(w > 0)$ is the selection intensity. Figure 5 depicts that there exist intermediate α values maximizing the fraction of cooperators, and there exists an upper bound value of R beyond which cooperators go extinct. This indicates that our findings are robust against the changes of update rule. Moreover, the upper bound value of R is much higher than the one determined under the imitation rule. In fact, under birth-death update rule at each time step the amount of common resource in each group is updated, but only one individual is chosen for updating the strategy. Thus, the amount of common resources is updated faster than individual strategies, and the upper bound thus becomes larger.

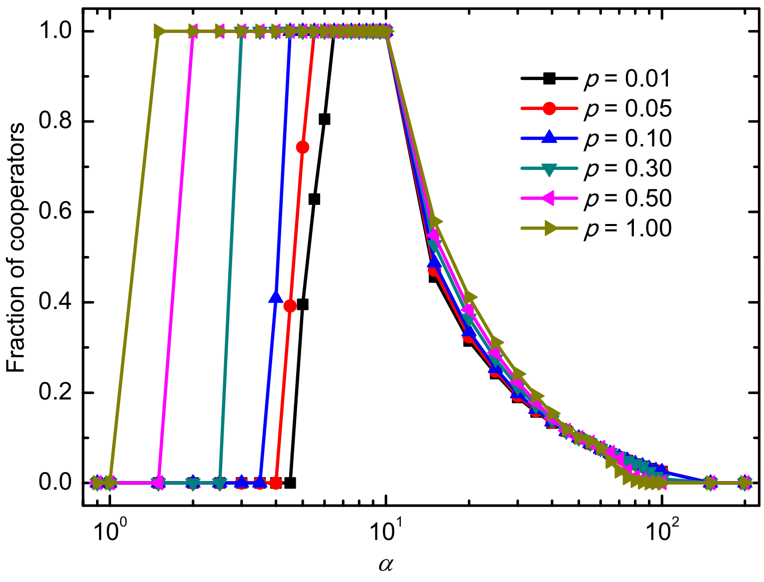

FIG. 6: The stationary fraction of cooperators in dependence on the multiplication factor α for different probability values of cooperator's priority p. Other parameter values are: $b = 10$, $K = 0.5$, $R = 10$, and the size of the square lattice is $L^2 = 10^4$.

Cooperator's priority towards limited endowments

Inspired by Ref. [5], in this section we consider that cooperators can have the priority to use the common resource they produced, especially when the common resource is limited. To be specific, when the common resource is not abundant, cooperators can preferentially to obtain an endowment at a certain probability p. Then, a cooperator's endowment from group i is given as

$$a_c^i = \begin{cases} b & \text{if } n_c b \leq R_i(t) < Gb, \\ R_i(t)/n_c & \text{if } 0 < R_i(t) < n_c b, \end{cases} \quad (1)$$

and a defector's endowment from the same group is given as

$$a_d^i = \begin{cases} \frac{R_i(t)-n_c b}{G-n_c} & \text{if } n_c b \leq R_i(t) < Gb, \\ 0 & \text{if } 0 < R_i(t) < n_c b, \end{cases} \quad (2)$$

where n_c is the number of cooperators in group i. With probability $1-p$, an individual's endowment is assigned according to the method in the main text. In Fig. 6, we show that there still exists an intermediate value of the multiplication factor inducing the maximal fraction of cooperators for different value of p, when the initial amount of common resource is limited. In addition, increasing the p value can further favor the evolution of cooperation. This is because, if cooperator's priority

towards limited common resource is amplified, cooperators simply obtain more opportunities to collect a higher payoff than defectors.

[1] Szabó, G. and Fáth, G. Evolutionary games on graphs. *Phys. Rep.* **446**, 97–216 (2007).

[2] Perc, M., Gómez-Gardeñes, J., Szolnoki, A., Floría, L. M., and Moreno, Y. Evolutionary dynamics of group interactions on structured populations: a review. *J. R. Soc. Interface* **10**, 20120997 (2013).

[3] Traulsen, A., Shoresh, N., and Nowak, M. A. Analytical results for individual and group selection of any intensity. *Bull. Math. Biol.* **70**, 1410–1424 (2008).

[4] Rand, D. G., Rarnita, C. E., Ohtsuki, H., and Nowak, M. A. Evolution of fairness in the one-shot anonymous ultimatum game. *Proc. Natl. Acad. Sci. USA* **110**, 2581–2586 (2013).

[5] Gore, J., Youk, H., and van Oudenaarden, A. Snowdrift game dynamics and facultative cheating in yeast. *Nature* **459**, 253–256 (2009).
